# Evolution of Scientific Production on Health Literacy and Health Education—A Bibliometric Analysis

**DOI:** 10.3390/ijerph19074356

**Published:** 2022-04-05

**Authors:** Laia Selva-Pareja, Anabel Ramos-Pla, Pere Mercadé-Melé, Anna Espart

**Affiliations:** 1 Faculty of Nursing and Physiotherapy, University of Lleida, 25198 Lleida, Spain; anna.espart@udl.cat; 2 Department of Nursing and Physiotherapy, University of Lleida, 25198 Lleida, Spain; 3 Health Education Research Group (GREpS), University of Lleida, 25198 Lleida, Spain; 4 Càtedra de Desenvolupament i Territoris Saludables (DOTS), University of Lleida, 25001 Lleida, Spain; anabel.ramos@udl.cat; 5 Department of Pedagogy, Faculty of Education, Psychology and Social Work, University of Lleida, 25001 Lleida, Spain; 6 Organisational Development Team (EDO-UdL), University of Lleida, 25001 Lleida, Spain; 7 Department of Statistics and Econometrics, University of Malaga, Andalucia-Tech, SEJ-645, 29071 Málaga, Spain; 8 Health Care Research Group (GRECS), Lleida Institute for Biomedical Research Dr. Pifarré Foundation, IRB Lleida, 25198 Lleida, Spain

**Keywords:** health literacy, health education, bibliometric analysis, VOSviewer, RStudio, machine learning

## Abstract

In the last few years, there has been an emphasis on the importance of health literacy (HL) and health education (HE) as basic tools to empower individuals and the community. The increasing interest in HL and HE has been observed through the evolution of publications and the nature of the main trends in the last few years. Knowing how HL and HE have evolved in scientific publications can help us to identify trends and set work priorities in this scope. Based on this, a bibliometric analysis (from 2000 to 2021) was conducted in two phases: first, an analysis was performed on the publications included in the Web of Science (WOS); second, a more specific analysis was conducted on the Core Collection from WOS. The data were analyzed with two software programs, the and Bibliometrix package for RStudio, and VOSviewer to analyze number of publications, citations, authors, collaborations, keywords trends, keywords evolutions and clusters of related terms. A total of 1799 articles were found in the first phase, and 727 in the second. The results from both analyses showed that the publications increased unequally until 2020, and considerably decreased in 2021; however, in spite of this, the number of citations remained constant. Likewise, five word clusters related with HL and HE were identified. D. Nutbeam stood out as the most prolific author on the subject, the USA as the country with the most publications, and the International Journal of Environmental Research and Public Health as having the most articles on the subject. This analysis may be a useful and helpful tool for future studies on the subject.

## 1. Introduction

The origins of the concept of Health Literacy (HL) go back to 1974, when S.K. Simonds associated it with another closely-related concept, Health Education (HE) [1]. Both concepts were defined in the area of school education, and it was not until 1998 that the World Health Organization (WHO) adopted HL as a concept also in the area of public health, defining it as “cognitive and social skills which determine the motivation and ability of individuals to gain access to, understand, and use information in ways which promote and maintain good health” [2]. Posteriorly, the WHO further developed this concept, introducing nuances which defined the objective of HL as “to take action to improve personal and community health by changing personal lifestyles and living conditions” [3]. Starting with this definition, the WHO highlighted that HL is fundamental for the personal, social, and cultural development of individuals, and could therefore have an influence on the health of individuals, in a critical manner for health empowerment. At the same time, HE was defined as the “opportunities for learning involving some form of communication designed to improve HL, including improving knowledge, and developing life skills which are conducive to individual and community health” [4]. In this sense, HE promotes HL, given that it is the education process of the population in general, and that of each individual, which promotes changes towards healthy behaviors [5,6]. Therefore, both concepts are encompassed within the concept of health promotion, with it being a necessity, and at the same time a priority, especially in health and school systems, as a pillar in the maintenance and improvement of health conditions [7,8].

Likewise, delving further into the framework of the European Health Literacy Project, HL also contemplates the competences of individuals in understanding, evaluating and applying health information to maintain and improve the quality of life [9].

### 1.1. Health Literacy and Its Construction as a Concept

There are three levels in HL: (i) functional: basic competences of reading and writing to act efficiently in a health context; (ii) interactive: cognitive and social skills that allow for active participation in subjects related to health; and (iii) critical: competences associated with the making of decisions starting with a critical analysis and the use of information to participate in health actions [10,11]. These three levels articulate the degree of knowledge acquired by each individual as a function of age, culture, and prior knowledge, among others.

Aside from these three levels, there are three dimensions based on a conceptual model with respect to HL, and which must be considered to ensure the correct literacy in this area: attention and care; prevention of diseases, and health promotion. Starting with these three dimensions, we find four more, which refer to the processing of information, based on the logical model: accessing, understanding, processing, and application of the information received about health subjects [5,6,9,12,13,14]. The Sørensen model combines these two models and creates a new integrated model in which other factors are considered, such as determinants (social and environmental, situational, and personal), or the course of life, so that social, anthropological, and psychological approaches can be established around the concept and application of HL [14].

### 1.2. Health Literacy and Its Relevance Today

The interest of the general population and the need to implement real HL in education contexts has increased, given its high impact on health results, in which a greater HL is associated with a better state of health. In this sense, many studies [15,16,17] have stated that a greater HL, along with a greater empowerment, improves the competence of decision-making, and therefore, the individual acquires a more active role in the process. Diverse authors have pointed out that the different health literacy programs or projects, which were mainly conducted in Compulsory Secondary Education, improved the results regarding health knowledge and emotional well-being, and empowered students to seek help when they needed it [18,19,20].

The health crisis due to COVID-19 has further highlighted, if possible, the need for HL through the implementation of HE, given that the general population has had to rapidly learn and apply a set of health measures and protocols to contain the expansion of SARS-CoV-2 [21,22,23].

The evolution of HL and HE concepts has become consolidated in the last few decades. Thus, the objective of this review is to conduct a quantitative bibliometric analysis to discover what the advances and changes have been with respect to HL and HE in the last few years. The results obtained will allow us to identify the trends and priorities in this field, as this method is efficient and effective for quantitatively describing the influence of a subject through time [24,25,26,27].

## 2. Materials and Methods

### 2.1. Data Source and Collection

The bibliometric analysis was performed with the Web of Science (WOS) database, due to their well- and deeply cited interconnections in several research areas through the Web of Science Core Collection (WOSSC), allowing us to perform a precise and specific analysis of publications, authors, citations, and keywords. For this, a search was conducted for articles which included the subject TS = (“health literacy” and “health education”). The inclusion criteria were: (a) articles published between 1 January 2000 and 31 December 2021, and (b) original articles in any language, and the exclusion criteria were: (c) any type of articles not considered originals (i.e., any type of reviews, meta-analyses and other documents such as books, letter to editor, editorial material, and similar). To ensure the inclusion of all articles and avoid problems with database updates, the search was completed in a single day on 31 December 2021. The screening according to the inclusion and exclusion criteria was performed in two phases. The first phase directly utilized the filters offered in the database, and the second was performed with the article’s titles. The search and selection results are shown in the Section 3.2., with a flow diagram adapted from the Preferred Reporting Items for Systematic Reviews and Meta-Analyses (PRISMA) [28].

The search for articles to be analyzed was conducted in two phases. In the first one, we worked with WOS “All Databases”, the searches of which are based on seven databases, and whose results provide a general view of all recorded documents in them. The results obtained in the first phase allowed us to identify the database WOSCC as the most appropriate for the second phase of analysis. For this reason, in the second one, we performed a more precise bibliometric analysis using WOSSC, where keywords, citations or authors were analyzed through their occurrence or as a cluster of concepts using two specific software for this purpose: Bibliometrix 3.1.4 for RStudio and VOSviewer.

### 2.2. Data Extraction and Study Selection

Likewise, a double screening was performed, the first with “All Databases”, and the second, more specific one with the WOSCC database, for a more in-depth bibliometric analysis. In both cases, the following information was extracted: year of publication, number of citations, authors, journals, areas of research, countries, languages, and keywords.

For the selection of studies, two of the authors (LS and AE) independently evaluated the articles. Any disagreement in the selection of articles was resolved via consensus with the third author (AR) of the present article.

### 2.3. Data Analysis and Visualization

The statistical analysis was performed with two specific programs, the “Bibliometrix 3.1.4” package [29] of RStudio (version 2021.09.1, RStudio Team, Boston, MA, USA) and VOSviewer (version 1.6.17, Leiden University Center for Science and Technology Studies, Leiden, The Netherlands) [30], both of which were developed to perform bibliometric analyses. The Bibliometrix package allows quantitative calculations of the items included in the analyzed articles. In turn, both software allow the construction and visualization of bibliometric networks to facilitate the understanding of this type of studies [31,32]. Specifically, the RStudio software program, through the package “Bibliometrix 3.1.4” for the application of Machine Learning, evaluates the distribution of each component analyzed in the bibliometric analysis. For this, the following variables were utilized: top authors’ production over time, historical direct citation network (historiography with maximum 20 nodes), source growth (occurrences cumulate, >5 number of sources), source local impact by H index (>5 of sources), most local cited sources, country of scientific production, most relevant affiliations, most cited countries, countries’ collaboration networks and most relevant words and trend topics (KeyWords Plus and author’s keywords, with three words per year and ten words minimum frequency). At the same time, a Multiple Correspondence Analysis (MCA) was performed with categorical data through the use of keywords from the authors and limiting the number of keywords to 50, to obtain different relationship clusters.

Likewise, VOSviewer was used to analyze and visualize the bibliometric networks of the keywords (50 keywords with frequency more than 20 occurrences). The aim of this co-occurrence analysis, through the application of the KeyWords Plus and author’s keywords, was to view the networks according to the number of citations and their temporality.

## 3. Results

The results are organized starting from a more general analysis (through the “All Databases”), to a more specific one (using WOSCC).

### 3.1. Search Results in All Databases

The search in All Databases provided 2353 initial records. Once the inclusion criteria and the selection of articles according to their titles were applied, a total of 1799 articles were selected. Of these, 84.04% were found in the WOSCC, 81.32% in MEDLINE^®^, 60.81% in Current Contents Connect, and 42.52% in CABI: CAB Abstracts. The other databases contained in WOS only included less than 5% of the articles found in this search.

#### 3.1.1. Publication Year, Citation Count and Authors

**Figure 1:**
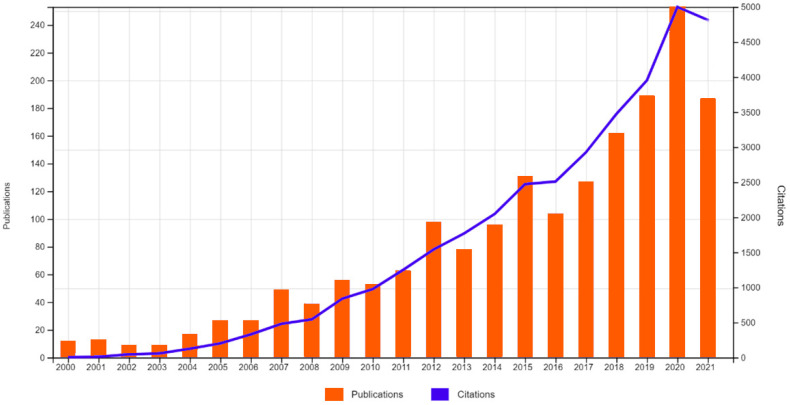
Number of publications and citations per year. Source: Web of Science (adapted).

Despite the number of articles published increasing since the year 2000, this increase was not uniform, and it was observed that some years (i.e., 2012 or 2015) were more productive that the years right after. The year with the most publications was 2020, with a total of 253 articles (Figure 1).

With respect to the number of citations of the articles included in the analysis, these were cited 35,455 times, 32,932 when excluding self-citations. The maximum number of citations in a single year was in 2020 (5000 citations), coinciding with the greater number of publications. The five most productive years were, from most to least, the following: 2020, 2019, 2021, 2018, and 2015 (see Table 1).

With respect to the most prolific authors in the period studied, the top five were: A.F. Jorm and M.S. Wolf with 13 publications each, D. Nutbeam and D.W. Baker with 11 publications, and T.C. Davis with 10 publications.

#### 3.1.2. Journals, Research Areas, Countries and Language of Publication

With respect to the journals with the greatest number of publications on HL and HE, the following stood out, according to number of publications: International Journal of Environmental Research and Public Health (*n* = 53, 2.95%), Journal of Health Communication (*n* = 47, 2.61%), BMC Public Health (*n* = 39, 2.17%), Patient Education and Counseling (*n* = 28, 1.56%) and Health Promotion Practice (*n* = 23, 1.28%).

Regarding to the areas of research in which the most articles were published, the following were underlined: Public Environmental Occupational Health (*n* = 693, 38.52%), Health Care Science Services (*n* = 391, 21.73%), Education Educational Research (*n* = 266, 14.79%), Psychology (*n* = 161, 8.95%) and Nursing (*n* = 140, 7.78%).

Lastly, with respect to the countries that published the most on the subject, the following were found: the USA (*n* = 705, 39.19%), Australia (*n* = 186, 10.34%), China (*n* = 87, 4.84%), Canada (*n* = 81, 4.50%) and Germany (*n* = 78, 4.34%). Additionally, the most utilized languages used in these articles were: English (*n* = 1674, 93.05%), well ahead of Chinese (*n* = 55, 3.06%), with smaller percentages found for German, Korean, Portuguese, and Spanish.

### 3.2. Search Results in Web of Science Core Collection

**Figure 2:**
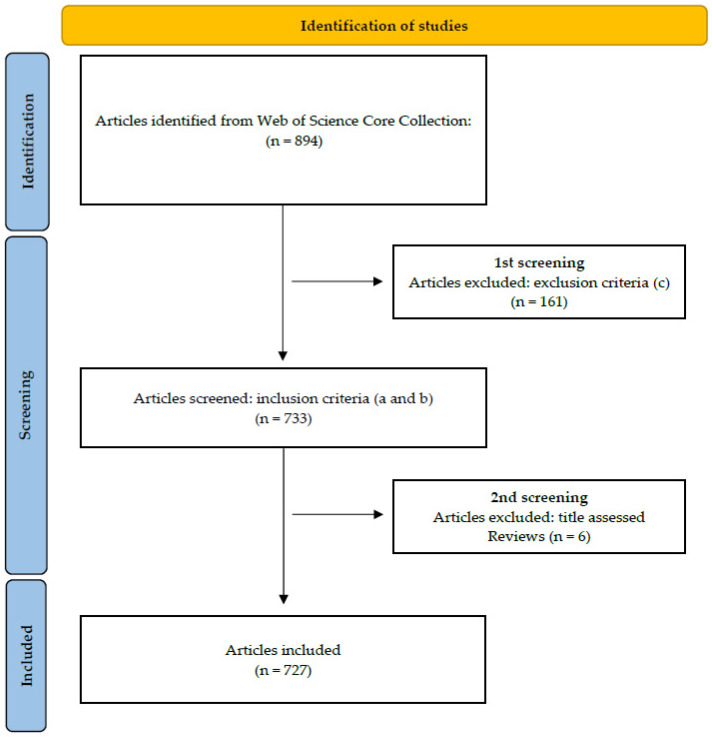
Flowchart of the results of the search according to the PRISMA standard (adapted version). (a–c): inclusion and exclusion criteria (see Section 2.1).

The search in the WOSCC database resulted in 894 records, of which, after the application of the inclusion and exclusion criteria, and after evaluating them according to the titles, 727 were included in the study. It should be underlined that despite these articles being found in the WOSCC database, a great many were also found in other databases such as MEDLINE (78.95%), Current Content Connect (66.99%), CABI:CAB Abstracts (29.16%), SciELO (2.61%) and KCI-Korean Journal Databases (0.14%) (Figure 2).

#### 3.2.1. Publication Year, Citation Count and Authors

**Figure 3:**
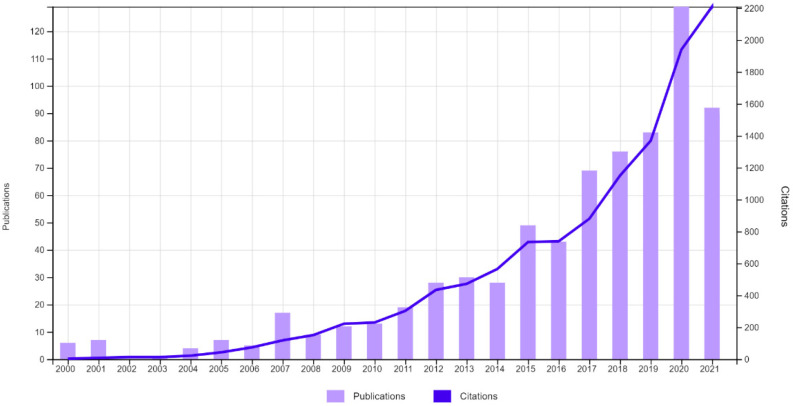
Number of publications and citations per year. Source: Web of Science.

The trend, with respect to publications and the number of citations, increased from the year 2000 until 2020, reaching a total of 129 publication and 2011 citations in the latter. On the contrary, the number of publications in 2021 decreased to 92 publications, although the number of citations was practically unchanged, with a total of 2002 (Figure 3).

As for the number of overall citations, the articles were cited 11,807 times, 11,194 times excluding the self-citations. The top five articles cited accumulated a total of 3573 citations (mean = 714.6). The two most cited articles [33,34] corresponded to the same author, D. Nutbeam. The years with the most citations were, from most to least, 2000, 2008, 2013, 2011, and 2001 (Table 2).

**Figure 4:**
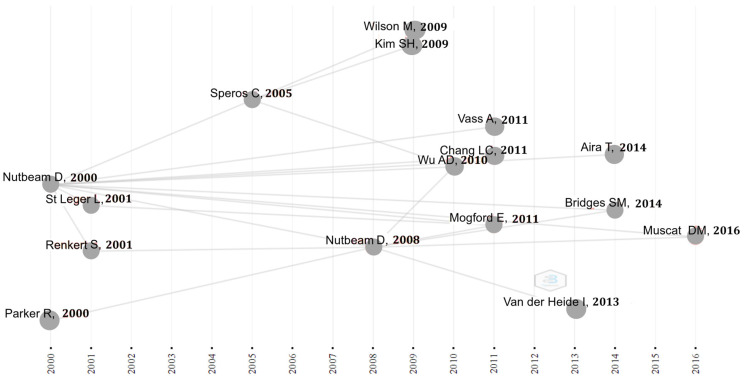
Historical direct citation network.

With respect to the authors who published the most between 2000 and 2021, we found D. Nutbeam (author of the two most cited articles [33,34] in the period analyzed), and L Paakkari (Table 3). Of the five authors with the most publications, we identified D. Nutbeam as the author with the greatest trajectory, who started to publish in the year 2000, while other authors, such as L. Paakkari, A. Arora, S.H. Kim and C.H. Liu, began to publish in the year 2009 (Figure 4).

As for the citations between authors throughout the period studied, it was observed that the promoter of HL and HE was D. Nutbeam, starting with his publication “Health literacy as a public health goal: a challenge for contemporary health education and communication strategies into the 21st century” in the year 2000 [33], and once again in 2008 with the publication “The evolving concept of health literacy” [34]. Both articles have been used as references for other publications from other authors. In this way, as shown in Figure 4, the first publication of D. Nutbeam appeared in the year 2000 on this topic is the main source of citation of several authors during the following sixteen years. Another publication of this author from the year 2008 is also used as an important source of reference, though to a lesser extent, in publications from 2011 to 2016. Likewise, other more recent cited authors (e.g., C. Speros, 2005; A. D. Wu, 2011; or E. Mogford, 2011) had previously cited D. Nutbeam (Figure 4).

#### 3.2.2. Journals and Research Areas

**Figure 5:**
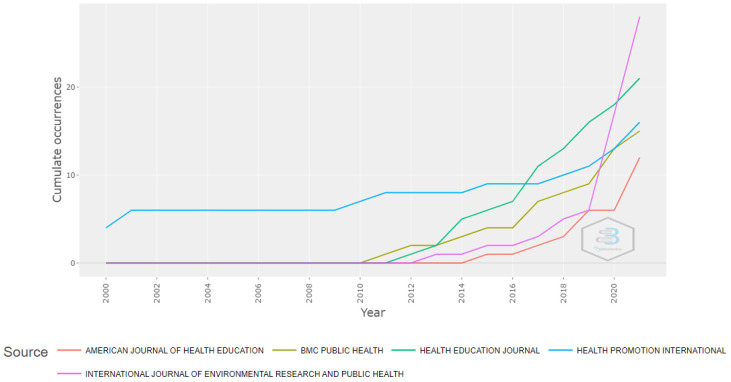
Source Growth.

The journals which published the most on the subject were: the International Journal of Environmental Research and Public Health (3.95%), Health Education Journal (2.89%), Health Promotion International (2.20%), BMC Public Health (2.06%) and American Journal of Health Education (1.65%) (Table 4). The first journal to publish on the subject was Health Promotion International, but the journal that has experienced the greatest growth in the last few years is the International Journal of Environmental Research and Public Health (Figure 5). As for the H-Index, the journal with the greatest impact was Health Promotion International (H-Index = 9), followed by Patient Education and Counseling (H-Index = 8), BMC Public Health and International Journal of Environmental Research and Public Health (both with an H-Index = 7), and lastly, Health Education Journal (H-Index = 6).

As for the number of citations, the five most cited journals were: Patient Education and Counseling with 530 citations, BMC Public Health with 568 citations, Health Promotion International with 417 citations, the Journal of General Internal Medicine with 403 citations and the Journal of Health Communication with 364 citations.

According to Journal Citation Reports, the impact factor of the journals with the most publications were between 3390 and 1299, in four different areas of knowledge (Environmental Sciences; Public, Environmental and Occupational Health; Education and Educational Research; and Health Policy and Services), which were all classified between the Q1 and Q4 quartiles (Table 4).

From the WOS analysis, it was found that the top five research areas, according to the number of articles, were: Public, Environmental and Occupational Health (44.15%, *n* = 321); Health Care Science Services (13.89%, *n* = 101); Nursing (11.55%, *n* = 84); Education Educational Research (10.73%, *n* = 78); and General Internal Medicine (7.70%, *n* = 56).

#### 3.2.3. Countries of Origin and Language

**Figure 6:**
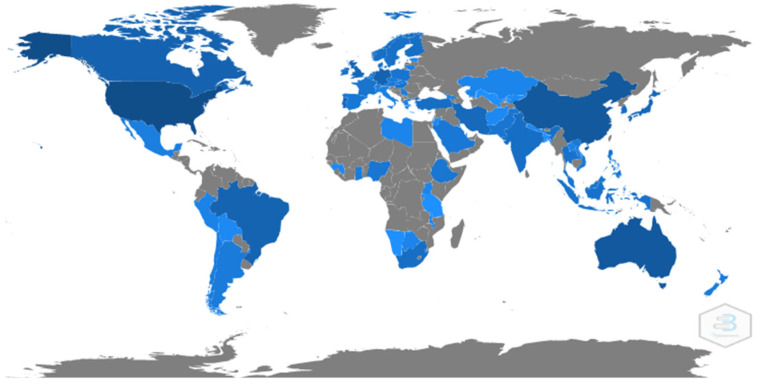
Countries with the highest scientific production in HL and HE. The more intense the blue, the greater the contribution to scientific production. In grey, countries without scientific production identified in this topic.

The countries with the most publications on the subject were: 42.64% USA, 11.55% Australia, 8.11% People’s Republic of China, 5.64% Germany, and 5.09% Canada (Figure 6).

**Figure 7:**
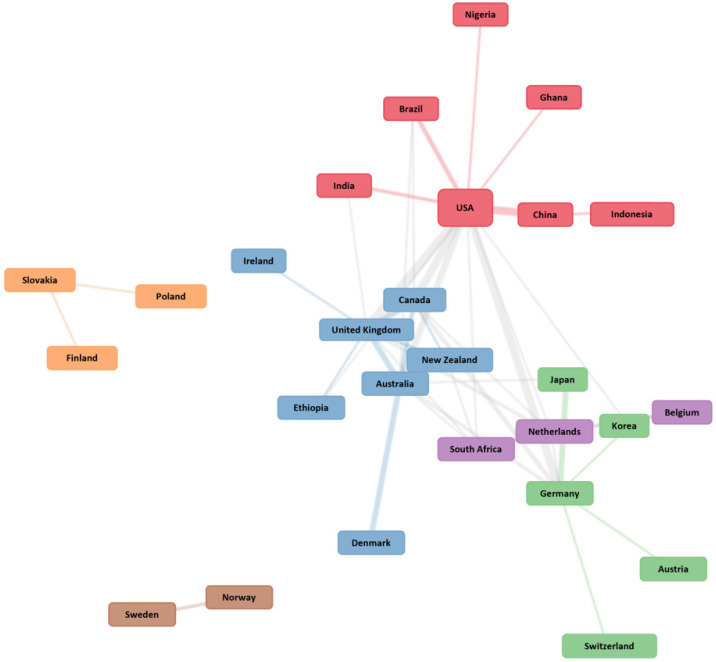
Countries’ collaboration network. Each color indicates a specific cluster and their relationship within or with other clusters.

As for the collaboration between countries, six clusters were identified, two of which (Slovakia–Poland–Finland and Sweden–Norway) did not establish a relationship with other countries that were not part of their same cluster, while another four were also found which did not have a relationship between countries within the same cluster, but instead had a relationship with the other three clusters (Figure 7).

With respect to the affiliations of the authors, the University of Sydney (Australia) was identified as the one which possessed the greatest number of publications with 59 articles, followed by the University of California—including all campuses (USA) with 43 articles, and Columbia (USA) and Emory (USA) universities, with 32 publications each. As for the countries with the most citations, among the top ten, we identified: USA with 4256 citations, Australia with 3968, China with 703, Canada with 508, the Netherlands with 337, United Kingdom with 203, Germany with 197, Brazil with 139, Finland with 127 and New Zealand with 89.

As for the language of the publication, 96% (*n* = 698) were published in English, 1.38% (*n* = 10) in German, and with a percentage of less than 1%, from higher to lower: Portuguese, Spanish, Chinese, French, Hungarian, Malay, and Turkish.

#### 3.2.4. Keywords

**Figure 8:**
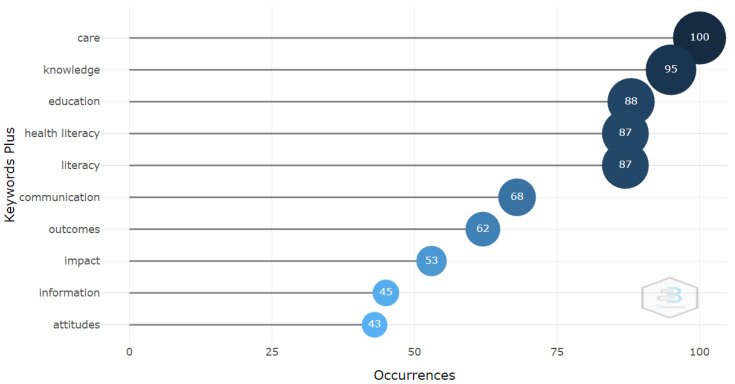
Most relevant KeyWords Plus.

With respect to the keywords, considering the most often repeated 10 KeyWords Plus within the 727 articles analyzed, the five most repeated words were: care, knowledge, education, health literacy and literacy; with 100, 95, 88, 87 and 87 repetitions, respectively (Figure 8). Among the keywords provided by the authors, the most often repeated were health literacy with 341 repetitions, and health education with 245 repetitions.

**Figure 9:**
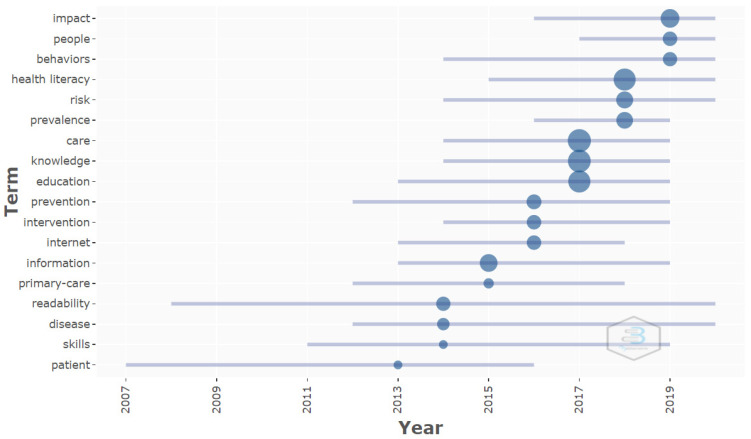
Trend topics authors’ KeyWords Plus.

**Figure 10:**
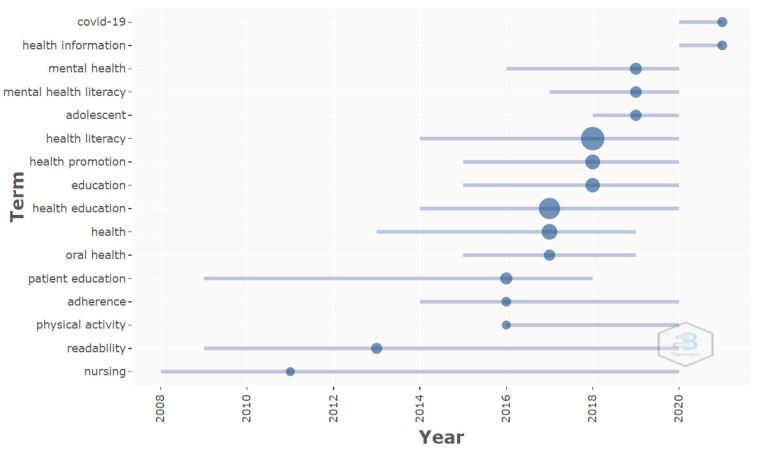
Trend topics authors’ keywords.

Through the analysis of the trend topics, starting with the selection of the KeyWords Plus and the author’s keywords with a minimum occurrence of 10, and by selecting a maximum of three keywords for each year analyzed, it was observed that the KeyWords Plus used in the 2017 were the most often repeated. These were: care, knowledge and education, with a frequency of 100, 95 and 88 co-occurrences, respectively. The keyword health literacy was utilized most in 2018, with co-occurrence of 87 times. With respect to the author’s keywords, health literacy was the most utilized, with a co-occurrence of 345 for 2018, while health education was the most repeated in 2017, with a total of 245 appearances (Figure 9 and Figure 10).

**Figure 11:**
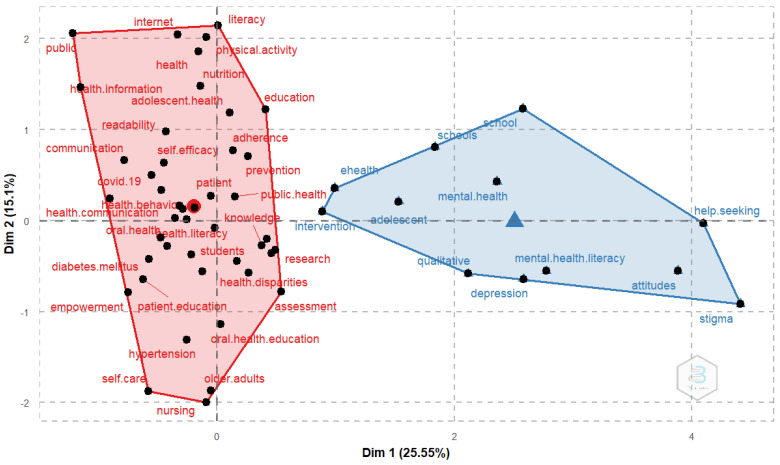
Conceptual structure map of authors’ keywords from the multiple correspondence analysis.

The MCA with the keywords from the authors showed two clusters, one of which was more concise, in blue (Figure 11), with the words school(s), ehealth, intervention, adolescent, mental health, help seeking, mental health literacy, qualitative, depression, attitudes and stigma, while the other one, in red, included a broad selection of terms that were more unrelated, such as: internet, health, communication, COVID-19, prevention, knowledge, patient education, nursing, empowerment, among others (Figure 11).

**Figure 12:**
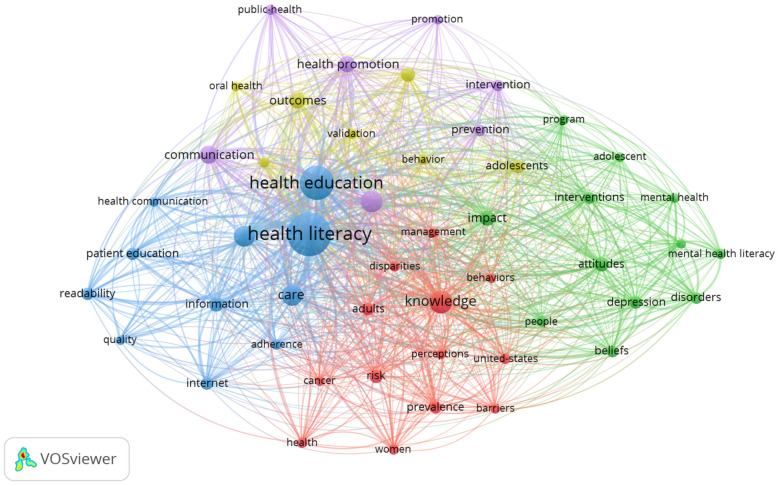
Network map of 50 keywords with a frequency of more than 20 occurrences.

Taking a step forward, after performing a co-occurrence analysis, in which the unit of analysis were all the keywords (KeyWords Plus and author’s keywords), with a minimum of 20 occurrences, a threshold of 50 was identified. In the resulting network map, it was observed that the ten words with a greater occurrence were health literacy (422 times), health education (263) knowledge (114), education (107), and care (100) (Figure 12).

Five clusters were underlined in this network, whose main concepts were:

- Cluster 1 (in red, 13 items): adults, barriers, behaviors, cancer, disparities, health, knowledge, management, perceptions, prevalence, risk, united-states and women.
- Cluster 2 (in green, 12 items): adolescent, attitudes, beliefs, depression, disorders, impact, interventions, mental health, mental health literacy, people, program and stigma.
- Cluster 3 (in blue, 11 items): adherence, care, health communication, health education, health literacy, information, internet, literacy, patient education, quality and readability.
- Cluster 4 (in yellow, 7 items): adolescents, association, behavior, children, oral health, outcomes and validation.
- Cluster 5 (in purple, 7 items): communication, education, health promotion, intervention, prevention, promotion and public-health.

The five clusters have a common node with HL and HE, with both terms acting as a hinge to the different subfields represented by each cluster. By delving deeper into these five clusters, some similarities are identified between clusters 1 and 3, including terms more focused on the concepts of illnesses, adultness, caring, and other terms related with diseases. Likewise, clusters 2 and 4 include terms related with adolescents and children, prevention, health knowledge and prevention of mental health issues. Finally, cluster 5 contains more general terms such as education, promotion, or prevention in health (Figure 12).

**Figure 13:**
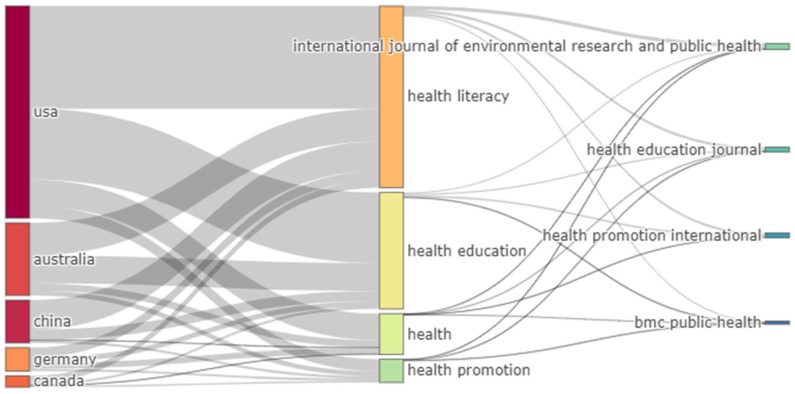
Summary plot of interactions between the most productive countries (**left**), the most relevant author keywords (**centre**) and the most relevant sources (**right**).

Lastly, a three-field plot was created, limited to five variables per column, in which the countries with the highest scientific production, the most important keywords, and the journals with the highest number of publications, were paired. It was observed that the countries with a greater number of publications mainly utilized four of the most relevant words (i.e., health literacy, health education, health and health promotion). These same words were included in articles published in four of the five journals that published the most, except for the journal Health Promotion International (Figure 13).

## 4. Discussion

The objective of this bibliometric analysis was to analyze the evolution and trends in the publications on HL and HE, and due to this, the two-phase analysis strategy allowed us to focus the results in a manner that was more specific and useful for obtaining reliable results. Since WOS and Scopus have similar characteristics in terms of bibliometric parameters that can be analyzed, we chose WOS because it allows a more robust evaluation of the documents housed in this database [38].

As far as we know, this is the first bibliometric analysis on HL and HE conducted at the international level. Although a similar study was published in 2008 [39], there are quite significant differences between the aims and results of both bibliometric analyses. While the study conducted by Kondilis analyzed research productivity (in GDP terms) and number of publications on selected fields related with HL (e.g., health perception or health competence) in European countries, our study sought to go further. Thus, we wanted analyze not only the world productivity (as number of scientific publications), but also establish the main interconnections between keywords, authors and countries. Furthermore, identifying trends in HL and HE through the years is also an interesting approach in bibliometric analysis that we wanted to explore, since it makes it possible to think about possible futures lines of action in this field. Another difference between both works is that while the Kondilis study is more focused on the term HL as a specific term, in our study, we worked with HL and HE as interconnected terms, as we consider them to be deeply linked to each other [39].

Finally, although some specific results could be comparable, such as number of publications per year, the development of new tools for bibliometric analyses allowed us to increase the number of results obtained, their complexity and the way they are displayed.

### 4.1. Publication Year, Citation Count, Authors, Journal and Research Areas

The searches carried out during each phase of the analysis identified uniform results for the number of publications. Thus, in both phases (i.e., analyses in All Databases and in WOSCC), an increase in the number of publications was observed from the year 2000 to 2020, and this increase was irregular in this 20-year period, with a decrease in the number of publications in specific years. In spite of this, there was a progressive and constant increase in the number of publications from 2016 to 2020, once again decreasing in 2021.

As for the number of publications that were most cited, D. Nutbeam was identified as the most cited author in the last 20 years on this subject, becoming the main axis for the rest of the authors who published articles on HL and HE. His two most cited articles [33,34] were published by two journals, Health Promotion International and Social Science and Medicine, respectively. These journals belong to the area of Public, Environmental and Occupational Health, with this being the area of research in which most of the HL and HE publications are found, which indicates that HL and HE belong to the area of public health due to their characteristics.

It must be highlighted that despite the Health Promotion International journal being the first journal to publish on this subject, the International Journal of Environmental Research and Public Health currently has the largest number of publications, and is also the one with the highest impact factor according to the Journal Citation Report.

As for the citations, the journals with the most citations related with HL and HE were Patient Education and Counseling and Health Promotion International. However, the most cited article, at present, was published in the Health Promotion International journal, which also had the highest H-Index compared to the other journals analyzed in this study. Likewise, of the top five cited articles, four of them were found in the category Public, Environmental and Occupational Health, which indicates that this category accumulates the highest number of citations on the subjects of HL and HE.

Lastly, even though D. Nutbeam was the most cited author, he did not have the highest number of publications on the subject, although he came in second place; this indicates that although D. Nutbeam was the promoter of the subject, as of today, there are other authors who have contributed more to the growth of this field of study.

### 4.2. Countries of Origin and Languages

With respect to countries and languages, the searches in the two phases coincided in that the USA was the country with the most publications (>39%), and English the most utilized, with 93% of the articles published in this language. These data were expected if the affiliations with the greatest number of publications are analyzed. Thus, the USA is the first country in terms of scientific contribution if we analyze the three universities with the most publications: University of California—including all campuses (USA), University of Columbia (USA), and Emory University (USA), while Australia is second; even though the University of Sydney publishes the most on the subjects of HL and HE, it does not exceed the number of publications from American universities.

At the same time, a set of countries that acted as central nodes in the network of collaborations were identified, when publishing articles on the subject. These were: USA, Canada, United Kingdom, Australia, New Zealand, South Africa, Netherlands and Germany. This indicates that the USA and Australia lead and channel the HL and HE subject, as shown by the number of citations they accumulate, which is higher than 1000 in both cases.

Unlike the study of Kondilis [39], focused on the European Union, the results shown here allow us to have a global vision where USA and Australia are, in fact, the most productive countries on this topic.

### 4.3. Keywords

The bibliometric analysis of the keywords conducted only through the WOSCC with the KeyWords Plus, the author’s keywords, and both together, indicated clear differences between the terms used, which is reasonable, given that the analysis was performed through the concepts health literacy and health education, so that the most relevant words utilized by the authors were these two, compared to the other terms. However, when analyzing the KeyWords Plus, meaning the keywords that are automatically generated from the titles of the articles cited, the most important words that were identified, from most to least, were: care, knowledge, education, health literacy and literacy, exceeding the concepts searched, care and knowledge, which suggests the non-existence of a correlation between keywords that the authors include in the section “keywords”, which are utilized to entitle their article. This indicates that the terms HL and HE are considered as general concepts which encompass other more specific terms, and are therefore the least utilized to define the issues that will be discussed in each of the articles published on the subject.

The analysis of the keywords through time revealed the same trend. Before 2016, the words were focused through the prism of disease and a nursing point of view, including KeyWords Plus such as patient, skills, disease, readability, primary care, information, internet and prevention; and author’s keywords such as: nursing, readability, physical activity, adherence and patient education. During the 2016–2018 period, words that were oriented towards education, health, and prevalence started to be used, with keywords such as knowledge, care, prevalence, risk, and health literacy, identified with KeyWords Plus, and oral health, health, health education, education, health promotion and health literacy as author’s keywords. Lastly, starting in 2019, the trend and direction changed, and HL and HE began to be associated with mental health, with KeyWords Plus such as: impact, people, behaviors, and as author’s keywords: adolescent, mental health literacy, mental health, health information and COVID-19. This change in the trend throughout the years demonstrates the versatility of HL and HE, as they adapt to the needs detected in health-related aspects.

This increase has been of such magnitude that the keywords associated with the subject have acquired a privileged position, as shown in Figure 10 (the keywords utilized by the authors), in Figure 11 (where a specific cluster was found related with mental health and adolescents), and also in how it also appeared specifically in one of the clusters from Figure 12.

In the case of mental health, the results obtained from the calculations performed in the study showed that this subject has become more important and relevant in the last few years, not only in terms of mental health as an issue, but also as a challenge in health literacy in specific groups, and in society in general [40,41].

Focusing on other more recent keywords such as COVID-19, up to 100 different publications from PubMed are retrieved, which include the terms COVID-19 and health literacy combined in their title. As several authors indicate, the situation of the COVID-19 pandemic has implied challenges not only in the diagnosis or treatment of this disease, but also in different areas such as health literacy [21,22,23]. One of the specific groups affected are adolescents, another relevant keyword on this study. Adolescents seem to be one of the most impacted groups in terms of mental health in the pandemic context, which would indicate that mental health literacy can be very useful as a strategy to avoid present and future challenges in this field [20,21,40,41,42]. In this way, some authors indicate a significant and positive relationship between HL and healthy behaviors in adolescents, but also in scholars, indicating the importance of applying HL and HE in educational centers to obtain a real impact on health. [18,19,20,23,42].

Lastly, and as a summary, a large homogeneity was found among the top five countries, author’s keywords, and journals. That is, the top five countries published articles about the four most important keywords (health literacy, health education, health and health promotion), and almost all of these appeared in the top five journals. It is interesting to highlight that given that the USA was the country with the most publications, these contained half of all the most relevant keywords, and only one of the five journals did not publish an article about one of the keywords; these are the Health Promotion International journal, and the keyword *health promotion*.

The main limitations of this study are that since it is a bibliometric analysis of two very broad concepts such as HL and HE, the results obtained may have yielded results that, though concise, do not allow the identification of specific relationships between HL, HE and other more specific concepts. In turn, this leads to another limitation: parameters such as transience index, m-Quotient or track citations and collaborations were not calculated, due to the large and variated number of terms related with HL and HE identified in the five different clusters (see Figure 12). Thus, for instance, calculating the transience index of publications with topics dealing with mental health, adolescents, cancer, stigma, oral health or women, among others, does not yield realistic and useful results in practice.

## 5. Conclusions

This is the first study in which a complete bibliometric analysis was conducted on Health Literacy (HL) and Health Education (HE) at the international level. As shown by the increasing number of publications, HL and HE have acquired, in the last two decades, a greater importance and relevance in the area of public health publications.

The number of publications over the last ten years indicates an expanding interest on this topic. Furthermore, the large number of terms closely related with HL and HE, identified in this study in five different clusters, shows the extensive scope that these two concepts have in health. The focus on what issues are relevant, in terms of HL and HE have evolved through the years going form aspects more related to illness, care and adultness, to others more closely to adolescents and children, prevention of mental health issues and even COVID-19. It can be perceived that, due to the challenges experienced in recent years with the COVID-19 pandemic situation, in the coming years the research in HL and HE will be relevant in mental health literacy. How to act, from the area of HE on vulnerable populations such as adolescents, how to increase knowledge on mental health management, how to modify their beliefs and attitudes or how to reduce the stigma on mental health will be the new challenges to face.

The results shown here can guide those who are approaching HL and HE for the first time, providing them relevant information to incentive the research in specific subfields on this subject.

HL and HE are very broad concepts covering many different keywords. Deeper bibliometric analyses are required, in which specific cluster of terms are considered, to learn more about differences in the evolution of scientific production on this field.

In any case, the information shown in this study is a useful tool that can stimulate new studies and collaborations between researchers from different areas and approaches within the HL and HE.

## Figures and Tables

**Table 1:** Number of publications and citations of last 10 years.

Year	No. Publications	Percentage of 1799 (%)	No. of Citations
2021	187	10.39	4813
2020	253	14.06	5000
2019	189	10.51	3953
2018	162	9.00	3474
2017	127	7.06	2927
2016	104	7.78	2510
2015	131	7.28	2473
2014	96	5.34	2047
2013	78	4.34	1770
2012	98	5.45	1541

**Table 2:** Most cited papers on Health Literacy and Health Education in Web of Science Core Collection (data from Web of Science).

Title	Authors	Year	Journal	Subject Area/Category	Citations (*n*)
Health literacy as a public health goal: a challenge for contemporary health education and communication strategies into the 21st century [33]	Nutbeam	2000	Health Promotion International	Health Policy and Services/Public, Environmental and Occupational Health	1873
The evolving concept of health literacy [34]	Nutbeam	2008	Social Science and Medicine	Social Sciences, Biomedical/Public, Environmental and Occupational Health	1144
The relationship between health, education, and health literacy: results from the Dutch Adult Literacy and Life Skills Survey [35]	van der Heide et al.	2013	Journal of Health Communication	Communication/Information Science and Library Science	254
The mechanisms linking health literacy to behavior and health status [36]	Osborn et al.	2011	American Journal of Health Behavior	Public, Environmental and Occupational Health	153
Schools, health literacy and public health: possibilities and challenges [37]	St Leger	2001	Health Promotion International	Health Policy and Services/Public, Environmental and Occupational Health	149

**Table 3:** The main authors who published the most on Health Literacy and Health Education in Web of Science Core Collection (data obtained from the Web of Science and VOSviewer).

Authors	No. of Papers	Percentage of 727 (%)	Citations (*n*)
Nutbeam, D.	8	1.10	3102
Paakkari, L.	5	0.69	65
Arora, A.	4	0.55	37
Kim, S.H.	4	0.55	71
Liu, C.H.	4	0.55	16
Ojio, Y.	4	0.55	36
Osborne, R.H.	4	0.55	50
Sasaki, T.	4	0.55	34
Togo, F.	4	0.55	34
Ando, S.	3	0.41	34

**Table 4:** Characteristics of the five journals with the most publications in the Web of Science Core Collection.

Journal Name	JIF (2020)	JIF without Self-Citations (2020)	Subject Area & Category	Edition	JIF Quartile (2020)	Number of Articles
**International** **Journal of** **Environmental** **Research and Public Health**	3.390	2.819	Environmental Sciences	SCIE	Q2	28
			Public, Environmental and Occupational Health	SSCI	Q1	
			Public, Environmental and Occupational Health	SCIE	Q2	
**Health Education Journal**	1.299	1.045	Education and Educational Research	SSCI	Q4	21
			Public, Environmental and Occupational Health	SSCI	Q4	
**Health Promotion** **International**	2.483	2.357	Public, Environmental and Occupational Health	SCIE	Q3	16
			Public, Environmental and Occupational Health	SSCI	Q2	
			Health Policy and Services	SSCI	Q3	
**BMC Public Health**	3.295	3.144	Public, Environmental and Occupational Health	SCIE	Q2	15
**American Journal** **of Health Education**	-	-	Public, Environmental and Occupational Health	ESCI	-	12

JIF: Journal Impact Factor; SCIE: Science Citation Index Expanded; SSCI: Social Sciences Citation Index; ESCI: Emerging Sources Citation Index.

## Data Availability

Data presented in this study are available on request from the corresponding author. The data are not publicly available due to the conditions of the project contract with the funder: DOTS University Chair (Chair for the Development of Healthy and Sustainable Organizations and Territories).
